# Decision-Making and the Alternative Five Factor Personality Model: Exploring the Role of Personality Traits, Age, Sex and Social Position

**DOI:** 10.3389/fpsyg.2021.717705

**Published:** 2021-10-27

**Authors:** Patricia Urieta, Anton Aluja, Luis F. Garcia, Ferran Balada, Elena Lacomba

**Affiliations:** ^1^Department of Psychology, Universitat de Lleida, Lleida, Spain; ^2^Lleida Institute for Biomedical Research (IRBLleida), Lleida, Spain; ^3^Department of Biological Psychology and Health, Autonomous University of Madrid, Madrid, Spain; ^4^Department of Psychobiology and Methodology of Health Sciences, Universitat Autònoma de Barcelona, Barcelona, Spain

**Keywords:** decision-making, conflict theory, Alternative Five Factor Personality Model, ZKA-PQ/SF, MDMQ

## Abstract

This study explores the relationship between decision-making style, as measured by the Melbourne Decision Making Questionnaire, and personality based on alternative five-factor model along with effect of age, sex and social position on such styles. A large sample of community and undergraduate students (*n* = 1,562; M_age_ = 40.03, *SD* = 18.43) was analyzed. The results showed that Neuroticism and Extraversion were significantly related to the non-vigilant styles Hypervigilance, Buck-passing and Procrastination. Women scored significantly lower in Vigilance and higher in Hypervigilance, Buck-passing and Procrastinations than men. Age was significantly related to decision-making style in a U-shaped fashion. The Social Position Index was significantly related to all decision-making styles. The most predictive personality domains regarding decision-making scales were Aggressiveness (negatively) and Activity for Vigilance, and Neuroticism for Hypervigilance, Buck-passing and Procrastination. Age, sex and social position had a small/medium overall effect on the four dimensions of Melbourne Decision Making Questionnaire (*p* < 0.001) with a η^2^ of 0.038, 0.068, 0.050, and 0.031 for Vigilance, Hypervigilance, Buck-passing and Procrastination, respectively. Based on scores on a single factor dimension of the MDMQ, the profile of participants with higher scores was characterized by lower age, more likely to be females, lower social position, higher levels of Aggressiveness, less Activity, less Extraversion, and higher Neuroticism.

## Introduction

Making decisions involves choosing a course of action, a possibility derived from a judgment about a problem or a condition that demands a choice and is characterized by personal beliefs about what resources can allow someone to achieve their own goals (Baron, 2008). Janis and Mann (1977) suggested a theory about conflict and decision-making. It assumed that decision-making entails a conflict that causes psychological stress due to concern about the serious personal, material and social losses that could be caused by the chosen alternative, and a concern about the loss of reputation and self-esteem if the decision goes wrong. The most remarkable symptoms of such conflicts are hesitation, vacillation, feelings of uncertainty, and signs of emotional stress when the decision becomes the focus of attention. Decision-making is complex in its nature and planning and decision-making processes depend on different variables that contribute to whether they work out successfully or not. In this way, decision-making has been related in previous studies to different individual and psychological variables such as age, sex and personality (Bouckenooghe et al., 2007; Heidari and Arani, 2017; Filipe et al., 2020). To a lesser extent, it has also been related to socioeconomic status (Sheehy-Skeffington, 2020).

### Decision-Making Assessment

Several self-reported questionnaires have been used in the field of decision-making research. Among them are the Rational Intuitive Decision Style Scale (RI-DSS) and the Proactive Decision-Making Questionnaire (PDMS) (Siebert and Kunz, 2016), but the most cited in the literature are the General Decision-Making Style (GDMS) (Scott and Bruce, 1995) and the Melbourne Decision-Making Questionnaire (MDMQ) (Mann et al., 1997). The MDMQ is a shortened 22-item version of the Flinders Decision-Making Questionnaire (Mann, 1982). This version measures coping patterns of decisional conflict and has been adapted to different languages and countries with satisfactory psychometric properties. For details, see the recent review by Filipe et al. (2020).

The shortened version of the MDMQ assesses decision-making styles according to four domains: Vigilance, Hypervigilance, Buck-passing and Procrastination. Vigilance refers to the way or style in which people seek objectives to make a decision by exploring alternatives, searching for rational and relevant information (e.g.,: I consider how best to carry out the decision). It also involves the assimilation of the information in an unbiased manner and the evaluation of different alternatives before making a decision. It results in a thorough information search, unbiased assimilation of new information, and other characteristics of high-quality decision making (Janis and Mann, 1977). The Hypervigilance style entails the frantic pursuit of decisions to get out of the dilemma, so decisions are made in a flash to relieve tension quickly. Hypervigilance includes emotional stress and limited perseverance (e.g.,: Even after I have made a decision, I delay acting upon it.). Buck-passing includes attribution of responsibility for one’s own decisions. Hesitation in decision-making is also related (e.g.,: I prefer that people who are better informed decide for me.). Finally, Procrastination means deferring decisions for later by lowering priority (e.g.,: Whenever I face a difficult decision, I feel pessimistic about finding a good solution.). Hypervigilance, Buck-passing and Procrastination are non-vigilant styles of decision making.

### Decision-Making and Personality

The MDMQ Vigilance scale shows positive correlations with the Five Factor Personality Model (FFPM) factors of Extraversion (Rahaman, 2014; Heidari and Arani, 2017), Agreeableness (Rahaman, 2014; Heidari and Arani, 2017), Conscientiousness (Di Fabio and Busoni, 2006; Rahaman, 2014; Heidari and Arani, 2017) and Openness (Di Fabio and Busoni, 2006; Di Fabio and Palazzeschi, 2012; Heidari and Arani, 2017), while these relationships are in the opposite direction for Neuroticism (Rahaman, 2014; Heidari and Arani, 2017). On the other hand, the three non-vigilant MDMQ scales have a significant positive relationship with Neuroticism (Vigilance; Rahaman, 2014; Procastination and Hypervigilance; Rahaman, 2014; Heidari and Arani, 2017). Indeed, the latter three MDMQ scales generally show opposite results to those observed for the Vigilance scale. For instance, a negative correlation with Extroversion has been reported for Buck-passing and Procrastination (Di Fabio and Busoni, 2006; Peter and Gurnáková, 2014; Rahaman, 2014), and Hypervigilance (Di Fabio and Busoni, 2006; Peter and Gurnáková, 2014; Rahaman, 2014; Heidari and Arani, 2017). This is also theoretically congruent with cognitive approach to personality since introverted-anxious people have an anticipatory bias toward threat signals, become more consciously and automatically aware of these threats, and focus attention on these threatening signals (Eysenck, 1992). The Buck-passing scale also shows negative correlations with Conscientiousness (Rahaman, 2014; Heidari and Arani, 2017) and Openness (Di Fabio and Busoni, 2006; Rahaman, 2014), although Peter and Gurnáková (2014) found it to have a positive relationship with the latter personality factor. Similarly, the Procrastination and Hypervigilance have shown a negative relationships with Agreeableness (Rahaman, 2014; Heidari and Arani, 2017), Conscientiousness (Di Fabio and Busoni, 2006; Rahaman, 2014; Heidari and Arani, 2017) and Openness (Di Fabio and Busoni, 2006; Di Fabio and Palazzeschi, 2012; Rahaman, 2014), although Peter and Gurnáková (2014) found a positive relationship between Hypervigilance and Openness.

With respect to the negative relationship that Neuroticism has with the Vigilance scale and the positive one it has with all the other MDMQ scales, research by Filipe et al. (2020) has shown that people with a vigilant decision-making style tend to be more satisfied with life, show positive affect, and better decisional self-esteem, conversely the remaining decision-making scales were associated with negative affect, lower satisfaction with life, and reduced decisional self-esteem (Filipe et al., 2020). Hence, the fact the Vigilant and Neuroticism are negatively related is congruent with the strong reverse association that has been observed between the Self-esteem and Neuroticism constructs (Judge et al., 2002; Aluja et al., 2007). Moreover, the role that anxiety has in making the actual choice process more difficult (Saka et al., 2008), also suggests that more neurotic individual should report less vigilant decision making styles (Eysenck, 1992).

As has been summarized, the relationship between MDMQ and personality has been the focus of multiple research studies, most of them involving the five-factor model. However, to our knowledge, there are no studies examining the relation of these decision-making styles with Zuckerman’s personality model (Aluja et al., 2010). This Alternative Five Factor Personality Model (AFFPM) includes domains such as Activity, Aggression and Sensation Seeking, whose relationship with decision-making is currently unknown. As already detailed, people who score higher in Neuroticism and lower in Extraversion (which are also factors of the Zuckerman’s model) are expected to score higher in Buck-passing, Procrastination, Hypervigilance and lower in Vigilance. In fact, the strong relationships with Decision-Making scales are expected to be found with Neuroticism. Further, given its relation to the Conscientiousness factor of the FFPM (García et al., 2012), Activity should be positively associated with Vigilance and negatively associated with Buck-passing, Procrastination, and Hypervigilance. Aggressiveness is negatively related to the Agreeableness factor of the FFPM, and, hence, should be positively associated with Buck-passing, Procrastination, and Hypervigilance. Sensation seekers are more attracted to risk, but tend to have less anxiety (García et al., 2012). Nonetheless, Zuckerman’s model could provide some light on individual differences in decision-making styles beyond that provided by the FFPM since Sensation Seeking (only considered as an Extroversion facet in the gold standard of the FFPM –NEO-PI-R) has previously been related to decision-making behaviors (Reynolds et al., 2019), especially with respect to unplanned and risky behaviors that have negative consequences for the person and the group (Kovács et al., 2017; Ioannidis et al., 2019). Young people are greater sensation seekers than older people, but no differences were found between young and old people when making risky decisions, so it is hard to hypothesize about the relationship between Sensation Seeking and MDMQ.

### Decision-Making, Sex and Age

Differences in decision-making have been found due to both age and sex, particularly with the MDMQ. Elderly people seems to be more reliant on emotion compared to experience and are also better at facing the emotional aspects of a problem (Löckenhoff, 2011; Filipe et al., 2020). Bouckenooghe et al. (2007) found that older people tend to score higher on Vigilance and younger people on non-vigilant styles of decision making. Sproten et al. (2010) examined the effects of aging on decisions in two domains of uncertainty: risk and ambiguity. No differences were found between younger and older participants when they make risky decisions, but when ambiguous situations were introduced, older subjects were less reluctant than the younger ones. On another study, adults showed higher competence and problem-solving ability than young people (Blanchard-Fields et al., 2004), whereas with respect to the MDMQ, the latter have comparatively higher values in Hypervigilance, Buck-passing, and Procrastination (Kornilova et al., 2018). Young adults were less capable than older adults of managing stress when making decisions, due to their higher levels of buck-passing, hypervigilance, and procrastination (Filipe et al., 2020). Sex differences have also been found. Females tended to score lower on Procrastination compared with males. Female and younger respondents scored higher on Hypervigilance compared with male and older respondents (Bouckenooghe et al., 2007).

### Decision-Making and Social Position

The social position (SP) of an individual together with the social role determines the place of an individual in the social environment and social organization. Low social position or socioeconomic status (SES) leads to problems and difficulties that affect health and generate social stress that can affect decision-making. People of low SP have poorer health than those with higher SP (Pamuk et al., 1998; Steenland et al., 2004). High SP is related to educational achievement, which provides better occupational opportunities and higher potential, enabling better nutrition, access to housing to health care resources (Winkleby and Cubbin, 2003). Chronic stress related to perceived social position may also predispose people with lower SP to illness (McEwen, 1998). Socioeconomic status has been related to biological indicators of physical and mental health status, such as heart rate, drowsiness, cortisol habituation to stress, body fat distribution (Adler et al., 2000), as well as self-reported results, such as depression (Demakakos et al., 2008) or perceived stress (Hamad et al., 2008).

Lower SP has also been related to poor decisions. People with lower income are more likely to be exposed to uncontrollable and controllable negative life events and tend to make bad decisions in economic reasoning (Bruine de Bruin et al., 2007). People with low social position tend to focus on immediate needs and make rational decisions in the proximate context of socioeconomic threat. These changes in psychological processes can hinder the achievement of long-term goals. Such people also struggle with making optimal decisions regarding health and finances (Sheehy-Skeffington, 2020).

### Aims of the Present Study

Age and sex have been related to decision-making, but no previous study separated age into ranges. Age ranges would allow a better understanding of the distribution of the relationship with decision-making. Although SP and decision-making has not received much study, there is evidence to suggest that people of different social class have different instrumental and social values, which influence decision-making (Wang and Tang, 2019). Analyzing these variables together with personality traits is important, since they may confound the relationship between decision-making and personality (Starcke and Brand, 2012).

Hence, the main objective is to explore the relationship between decision-making style and Zuckerman’s model of personality along with the examination of the role of age, sex and social position. From a practical point of view, locating the specific aspects of personality that contribute to difficulties in decision-making processes is highly relevant to improving such processes, and could help counselors and clinicians to overcome such difficulties. It seems unlikely that difficulties will be resolved without intervention focusing on the more chronic and dysfunctional personality antecedents of any decision-making problems.

## Materials and Methods

### Participants and Procedure

The sample consisted of a total of 1,562 participants (M_age_ = 40.03, *SD* = 18.43; 54.3% females). Males reported a slightly higher average age than females (42.16 vs. 39.06; *t-*test: 2.43; *p* < *0.025*). 556 participants (M_age_ = 21.23; *SD* = 8.85) were undergraduate students and 1,006 were community volunteers (M_age_ = 50.40; *SD* = 13.45). All participants were healthy Caucasian adults aged 18–88 years old and were recruited in the cities of Madrid and Lleida (Spain) by undergraduate students taking part in a university personality research and practice program. Students were asked to find one female and one male participant in each of the following five age ranges: 18–30 (39.1%), 31–40 (8.8%), 40–50 (17.7%), 50–60 (22.4%), and over 60 (12%) years old. Participants in the general population reported their education and occupational levels on a 1–7-point scale in order to obtain the Hollingshead Social Position Index (SPI; Hollingshead, 1957); [SPI score = (Occupation score X 7) + (Education score X 4)]. For the purpose of analyzing SPI, only the cases that provided SPI data were included, which constituted 64.4% of the total sample (Supplementary Table 1). Note that the highest SPI values correspond to the lowest SP, and that scores can be grouped into five SPI groups. All participants signed a standard informed consent document in accordance with the ethical guidelines of the university research committee.

### Measures

#### Melbourne Decision-Making Questionnaire

Melbourne Decision-Making Questionnaire (MDMQ; Mann et al., 1997). The MDMQ is a 22-item questionnaire used to assess decision-making style. It is a self-report inventory designed to measure the four main coping patterns identified in the conflict-theory model of decision-making (Janis and Mann, 1977): Vigilance (Vi), Hypervigilance (Hy), Buck-passing (Bp), and Procrastination (Pr). Participants indicated their agreement with statements on a 3-point Likert-type scale. Each item consists of three answers that are scored as follows: true (2), sometimes true (1) and not true (0). The Spanish version used in this research was validated by De Heredia et al. (2004). The 22 items support a 4-factor structure. For the present sample, the reliability coefficients were 0.79 (Buck-passing), 0.78 (Procrastination), 0.70 (Hypervigilance), and 0.74 (Vigilance).

#### Zuckerman–Kuhlman-Aluja Personality Questionnaire Shortened Form

Zuckerman–Kuhlman-Aluja Personality Questionnaire shortened form (ZKA-PQ/SF; Aluja et al., 2018). The complete ZKA-PQ contains 200 items, with 10 items per facet (Aluja et al., 2010). The short version has 80 items, 20-facets and 4 items per facet measuring five traits (factors): Aggressiveness (AG), Activity (AC), Extraversion (EX), Neuroticism (NE), and Sensation Seeking (SS). The response format is a 4-point Likert-type scale ranging from 1 (*strongly disagree*) to 4 (*strongly agree*). Validity and reliability evidence of the ZKA-PQ and ZKA-PQ/SF were presented in the original studies (Aluja et al., 2010, 2018) and in cross-cultural validations in various African, American, Asian, and European cultures and languages (Rossier et al., 2016; Aluja et al., 2020). The ZKA-PQ/SF and scoring keys are included in the Appendix of Aluja et al. (2018).

### Data Analysis

First, frequencies and percentages of sociodemographic and descriptive variables, alpha internal consistency, and inter-correlations were calculated. To study the relationship of the MDMQ domains with SPI and age, an ANOVA was performed comparing different groups of SPI and age, using Scheffe’s post-tests. Since the total sample was gathered from two populations, a separate factor analysis of the 22-item MDMQ using principal axis extraction and oblimin rotation method was computed for subjects from the community and university student populations. To test if the structure was stable across both samples, a Procrustes rotation and factorial congruence coefficients were performed. Goodness-of-fit indices were calculated for the factorial structure of the whole sample. Subsequently, the structure of the 20 facets of ZKA-PQ/SF and the 4 MDMQ domains were analyzed. To determine the predictive ability of ZKA-PQ/SF on the 4 MDMQ domains, a multiple regression analysis was performed. The analysis was performed using the “enter” and “stepwise” methods over the domains and facets, respectively. For the domains, the “enter” method was performed, but for the facets we applied the stepwise method with a more rigorous than usual criterion of PIN (probability of F to enter; *p* < 0.0001) and POUT (probability of F to remove; *p* < 0.10). To graphically visualize the predictive ability of the ZKA-PQ/SF, a single factor was extracted from the MDMQ and a LOESS (locally estimated scatterplot smoothing) regression was performed (Fox, 2000; O’Connor, 2005). This procedure is complementary to linear regression analyses and allows us to observe graphically how the personality domains (z-scores) are distributed across the general factor measured by the MDMQ. Statistical analysis was carried out using SPSS 25 (SPSS Corp., 2017) and Factor.exe (Ferrando and Lorenzo-Seva, 2017). Results files are available upon request from the first author.

## Results

### Sociodemographic Variables, Test Descriptive and Reliability

The frequencies and percentages per sex of all the participants and the sociodemographic data of the community participants were calculated (Supplementary Table 1). Means and standard deviations are also provided for age, SPI, the domains of personality, and decision-making questionnaires (Table 1). The reliability of ZKA-PQ/SF domains ranged from 0.81 to 0.90, and the reliability of the MDMQ between 0.71 and 0.78. The SPI mean was 34.01 (*SD* = 18.64), which corresponds to an average social position rating (32–47 range) (Table 1 and Supplementary Table 1).

**TABLE 1:** Mean, standard deviation, reliability, inter-correlations and sex differences.

	All subjects (*n* = 1,562)														
	M	SD	α	12	11	10	9	8	7	6	5	4	3	2	1
1. Age	40.02	18.43	−	−0.15	−0.11	−0.06	0.07	−**0.42**	−0.19	−0.15	0.07	−**0.20**	**0.20**	−0.06	

2. Sex	−	−	−	0.09	0.10	**0.21**	−0.06	−0.11	0.26	0.07	0.04	0.01	0.06		
*3. SPI (n* = *1,006)*	*34.01*	*18.64*	−	*0.15*	*0.20*	*0.18*	−*0.17*	−*0.10*	*0.18*	−*0.09*	*0.05*	*0.12*			

4. Aggressiveness	33.23	8.82	0.89	**0.20**	0.11	**0.23**	−0.18	0.18	**0.42**	−0.14	0.03				
5. Activity factor	41.81	7.29	0.81	−0.11	−0.13	−0.01	0.11	0.18	−0.04	**0.24**					
6. Extraversion	48.67	7.85	0.86	−**0.21**	−**0.22**	−**0.24**	0.06	**0.26**	−**0.29**						
7. Neuroticism	35.54	9.65	0.90	**0.41**	0.38	**0.57**	−0.10	0.03							
8. Sensation Seeking	37.44	8.83	0.85	0.03	−0.04	−0.10	−0.08								

9. Vigilance	9.28	2.20	0.74	−0.17	−0.15	0.07									
10. Hypervigilance	4.74	2.34	0.71	**0.47**	**0.41**										
11. Buck-passing	5.05	2.67	0.79	**0.46**											
12. Procrastination	3.21	2.34	0.78												

*M: Mean: SD: Standard deviation; SPI: Social Position Index. For correlations equal or higher ± 0.10 p-value < 0.001. Correlations equal or higher 0.20 in boldface (n = 1,562). SPI data correspond to 1,006 community subjects (italics).*

### Melbourne Decision-Making Questionnaire Factor Structure

The main aim of the present paper is not to analyze the factor structure of the MDMQ. However, since total sample was gathered from two different samples (community and students), is important to test if the structure was replicated in both samples. From both factor matrices, 4 factors were extracted with oblique rotation with the eigenvalue criterion one and Scree test. Parallel analysis also yielded 4 factors. The method to obtain the random correlation matrices was the permutation of the raw data (Buja and Eyuboglu, 1992). In both samples, all items were integrated into their corresponding theoretical factor. Both matrices were rotated to obtain a third matrix using the Procrustes method that also allowed us to calculate the Tucker factor congruence coefficients for the items and the factors (Supplementary Table 2). A value of congruence coefficients in the range [0.85 – 0.94] means that the factors from each sample compared display a fair degree of similarity, and a value greater than 0.95 is generally interpreted as the factors being practically identical (Lorenzo-Seva and ten Berge, 2006).

The overall congruence coefficient (0.98) indicates that both factor matrices were very similar. The factor congruence of the factors ranged between 0.97 and 0.99, and that of the items between 0.83 and 1 (Supplementary Table 2). To ascertain the goodness-of-fit indices, a Robust Unweighted Least Squares (RULS) analysis with robust Chi-square and Variance-scaled (Asparouhov and Muthen, 2010) and normalized direct oblimin rotation was performed. The Kaiser-Meyer-Olkin (KMO) test scored 0.89, and 4 factors were also extracted. The goodness-of-fit indices were: Non-Normed Fit Index (NNFI) 0.99; Comparative Fit Index (CFI) 0.99; Schwarz’s Bayesian Information Criterion (BIC) 1140.070; Root Mean Square Error of Approximation (RMSEA) – 0.03 and Root Mean Square of Residuals (RMSR) – 0.02.

### Decision-Making, Age, Sex and Social Position

Age was significantly and negatively correlated with Buck-passing (−0.11; *p* < 0.001) and Procrastination (−0.15; *p* < 0.001). Sex (categorical variable scoring 1 for male and 2 for female) correlated with Neuroticism (0.26; *p* < 0.001) and Hypervigilance (0.21; *p* < 0.001). SPI also correlated negatively with Vigilance (−0.17; *p* < 0.001) and positively with Hypervigilance (0.18; *p* < 0.001), Buck-passing (0.20; *p* < 0.001) and Procrastination (0.15; *p* < 0.001) (Table 1). We found moderate sex differences in age (*t*-test = −2.24; *p* < 0.03) and SPI (*t*-test = 0.19; *p* < 0.05), but with small effect sizes (Cohen, 1988). Males scored higher than females on Vigilance (*t*-test = −2.41; *p* < 0.02; *d* = −0.12) and females scored higher than males on Hypervigilance (*t*-test = 8.48; *p* < 0.001), Buck-passing (*t*-test = 3.99; *p* < 0.001) and Procrastination (*t*-test = 3.60; *p* < 0.001) with a Cohen’s *d* of 0.44, 0.20, and 0.18, respectively. The main personality differences between sexes were in Neuroticism (37.79 vs. 32.86), Sensation Seeking (36.57 vs. 38.46) and Extraversion (49.19 vs. 48.06) with an effect size of 0.53, −0.22, and 0.14, respectively (Table 2).

**TABLE 2:** Descriptive and sex differences.

	Female (*n* = 714)		Male (*n* = 848)						
	M	SD	M	SD	K	S	*t*-test	*p*<	*d*
Age	39.06	18.70	41.16	18.05	−1.04	0.27	−2.24	0.03	−0.11
SPI (W = 504; M = 502)*	35.18	19.20	32.84	18.00	−0.58	0.59	−1,98	0.05	0.13

Aggressiveness	33.34	8.90	33.11	8.73	−0.34	0.35	0.52	0.60	0.03
Activity factor	42.07	7.33	41.50	7.22	−0.10	0.08	1.54	0.12	0.08
Extraversion	49.19	7.78	48.06	7.90	−0.14	−0.36	2.83	0.01	0.14
Neuroticism	37.79	9.50	32.86	9.13	−0.58	0.14	10.40	0.001	0.53
Sensation Seeking	36.57	9.00	38.46	8.52	−0.46	0.12	−4.24	0.001	−0.22

Vigilance	9.15	2.23	9.42	2.15	0.99	−0.94	−2.41	0.02	−0.12
Hypervigilance	5.19	2.31	4.21	2.26	−0.38	0.17	8.40	0.001	0.44
Buck-passing	5.30	2.69	4.76	2.61	−0.10	0.37	3.99	0.001	0.20
Procrastination	3.40	2.39	2.98	2.25	0.01	0.64	3.60	0.001	0.18

M^:^ Mean; SD: Standard deviation; K: Kurtosis; S: Skewness; Cohen’s d:0.01: very small, 0.20: small, 0.50: medium, 0.80: large, 1.20: very large.* SPI is performed only form community subjects.

**FIGURE 1:**
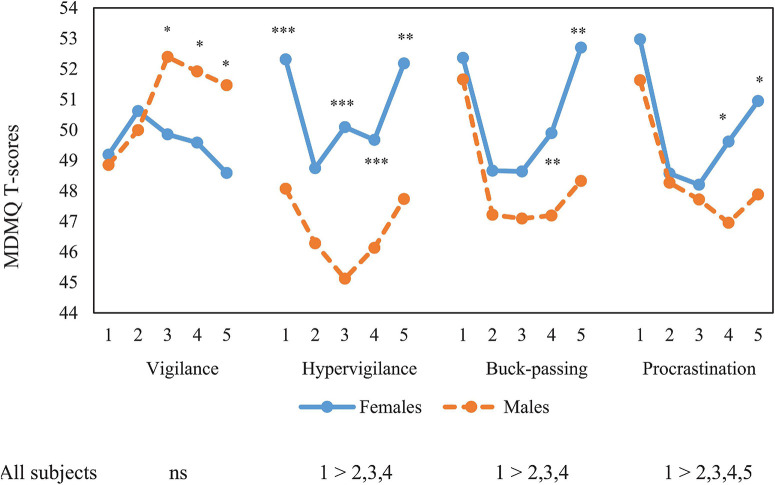
ANOVA mean co mparison of age ranges and MDMQ domains: 1: 30 years old and below; 2: 31–40 years old; 3: 41–50 years old; 4: 51–60 years old, and 5: more than 60 years old. Scheffe post-tests comparisons = *p* < 0.05. *T*-test sex comparisons * *p* < 0.05, ** *p* < 0.01, *** *p* < 0.001.

**FIGURE 2:**
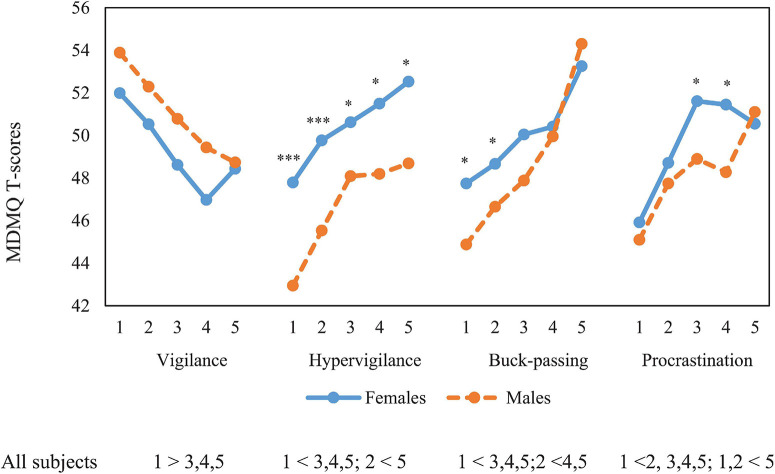
ANOVA mean comparison of SPI ranges and MDMQ domains: 1: Upper; 2: Upper-Middle; 3: Middle; 4: Lower-Middle and 5: Lower. Scheffe post-tests comparisons = *p* < 0.05. *T*-test sex comparison * *p* < 0.05, *** *p* < 0.001.

Figures 1, 2 show MDMQ scores for each of the age and SPI ranks, respectively, separately for males and females. No significant interactions between sex and either age or SPI ranges were observed for any MDMQ scale. Results when sex is included as a factor (and excluding SPI) show that Vigilance presented an inverted U-shaped distribution across age (*F*_(4,1)_ = 2.84; *P* = 0.023; η_p_^2^ = 0.007). Younger (under 30) and older (> 60 years old) age groups scored higher, but *post hoc* tests did not show significant differences between groups. In contrast, for Hypervigilance (*F*_(4,1)_ = 5.99; *P* < 0.001; η_p_^2^ = 0.015) and Buck-passing (*F*_(4,1)_ = 13.05; *P* < 0.001; η_p_^2^ = 0.033), a U-shaped distribution across age was observed, where young participants obtained significantly higher scores than middle-aged ones, but not for old-aged ones. A U-shaped distribution across age for Procrastination (*F*_(4,1)_ = 14.82; *p* < 0.001; η_p_^2^ = 0.037) was also observed. For this scale, young participants obtained significantly higher scores than the other age groups. We observed that from the age of 40 onward, males scored higher in Vigilance, females from the age of 51 onward scored higher in Buck-passing and Procrastination, and finally females scored higher in all age ranges in Hypervigilance. Apart from Vigilance, in both males and females there was a U-shaped distribution, i.e., young people under 30 and over 60 scored higher, while those in the intermediate ranges scored lower.

Similarly, with sex included as a factor, MDMQ Vigilance domain scores progressively dropped significantly with lower social position (*F*_(4,1)_ = 6.70; *p* < 0.001; η_p_^2^ = 0.026), unlike in the other three domains, Hypervigilance (*F*_(4,1)_ = 7.76; *p* < 0.001; η_p_^2^ = 0.03), Buck-passing (*F*_(4,1)_ = 10.59; *p* < 0.001; η_p_^2^ = 0.041) and Procrastination (*F*_(4,1)_ = 8.41; *p* < 0.001; η_p_^2^ = 0.033). Scores now increased progressively, and significantly, as SP decreased. Age, sex and social position had an overall significant effect on all four dimensions of the MDMQ (*p* < 0.001) with small and medium effects size (η^2^ = 0.038, 0.068, 0.050, and 0.031) for Vigilance, Hypervigilance, Buck-passing and Procrastination, respectively. Individually, age had a negligible or small effect on all four domains; sex had medium effect on Hypervigilance (η^2^ = ^0.063^) and SPI had a small effect for Buck-passing (η^2^ = 0.048). [η^2^ < 0.0099 = negligible; η^2^ > 0.01: small; η^2^ ≥ 0.0588 medium; η^2^ ≥ 0.1379: large effect size (Cohen, 1988, pp. 274–288)].

### Factor Convergence Between Decision-Making and Personality

To explore the relationship between the MDMQ domains and personality based on the Zuckerman’s model, we opted for a factor analysis of the 20 facets of the ZKA-PQ/SF together with the five MDMQ domains. We chose the principal axis extraction method with oblimin rotation. Five factors were extracted according to the structure of the ZKA-PQ/SF (Aluja et al., 2020). All facets of the ZKA-PQ/SF were integrated into their corresponding theoretical factor. The Vigilance domain of the MDMQ did not significantly load on any of the 5 personality factors. However, non-vigilant styles of decision-making. clearly loaded into the Neuroticism factor (Table 3).

**TABLE 3:** Factor analysis of ZKA-PQ/SF facets and MDMQ domains.

	I	II	III	IV	V
AG1 Physical Aggression ZKA	−**0.56**	0.13	−0.15	−0.11	0.01
AG2 Verbal Aggression ZKA	−**0.73**	0.01	0.00	0.14	−0.10
AG3 Anger ZKA	−**0.84**	−0.16	0.15	−0.02	0.07
AG4 Hostility ZKA	−**0.70**	−0.08	0.16	−0.09	0.01

SS1Thrill and Adventure Seeking ZKA	−0.01	**0.62**	−0.23	−0.10	0.13
SS2 Experience Seeking ZKA	0.09	**0.63**	0.06	0.14	0.07
SS3 Disinhibition ZKA	−0.05	**0.73**	0.13	0.26	−0.03
SS4 Boredom Suscep./Impulsivity ZKA	−0.04	**0.64**	0.03	−0.03	0.01

NE1 Anxiety ZKA	−0.27	0.01	**0.58**	−0.04	0.24
NE2 Depression ZKA	−0.13	−0.02	**0.73**	−0.03	0.00
NE3 Dependence ZKA	−0.12	−0.07	**0.73**	0.13	0.08
NE4 Low Self-esteem ZKA	0.00	0.04	**0.76**	−0.09	−0.07

EX1Positive Emotions ZKA	0.14	0.04	−0.23	**0.53**	0.13
EX2 Social Warmth ZKA	0.07	−0.08	−0.08	**0.56**	−0.08
EX3 Exhibitionism ZKA	−0.17	0.27	0.04	**0.54**	0.03
EX4 Sociability ZKA	0.03	0.10	−0.02	**0.78**	0.05

AC1Work Compulsion ZKA	0.01	0.07	0.06	−0.07	**0.45**
AC2 General Activity ZKA	0.03	0.10	−0.05	0.05	**0.72**
AC3 Restlessness ZKA	−0.25	0.20	0.07	0.05	**0.55**
AC4 Work Energy ZKA	0.08	−0.25	−0.16	0.18	**0.47**

*Vigilance*	*0.18*	−*0.13*	−*0.03*	*0.03*	*0.27*
*Hypervigilance*	*0.02*	*0.12*	***0.70***	−*0.02*	*0.11*
*Buck-passing*	*0.12*	*0.05*	***0.51***	−*0.07*	−*0.10*
*Procrastination*	*0.05*	*0.10*	***0.53***	−*0.07*	−*0.09*

*ZKA-PQ/SF: Zuckerman-Kuhlman-Aluja Personality Questionnaire shortened form; MDMQ: Melbourne Decision Making Questionnaire. Absolute value loadings equal or higher 0.30 in boldface.*

*MDMQ domains and loadings are in italics.*

### Personality Differences in Decision-Making Extreme Groups

**FIGURE 3:**
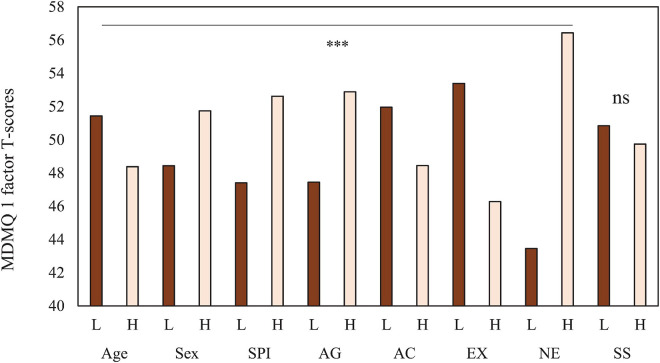
Age, Sex, SPI and personality *t*-test differences between low (30 percentile) and high (70 percentile) scores in MDMQ 1-factor. *** *p* < 0.001. L: Low and H: High. MDMQ 1 factor score.

One way to study the relationships between sociodemographic variables and personality and decision-making is to compare extreme groups on a single decision-making factor. For this purpose, we developed a variable with the factor loadings of the 22-item MDMQ obtained on a single non-rotated factor. Note that for this factor Vigilance items loaded negatively, so, this factor represents a non-vigilant decision-making style. Two groups were subsequently formed according to the 30th and 70th percentile: (a) subjects who scored low, and (b) subjects who scored high. The mean difference *t*-test was calculated for the sociodemographic and personality variables. The results are shown in Figure 3. For visual comparison, scores were transformed into T-scores. The results indicated statistically significant differences in all variables except Sensation Seeking. Participants with higher non-vigilant decision-making scores were young, mostly female, lower socially positioned, more aggressive, less active, more introverted, and highly neurotic (*p* < 0.001).

### Personality as a Predictor of Decision-Making

**FIGURE 4:**
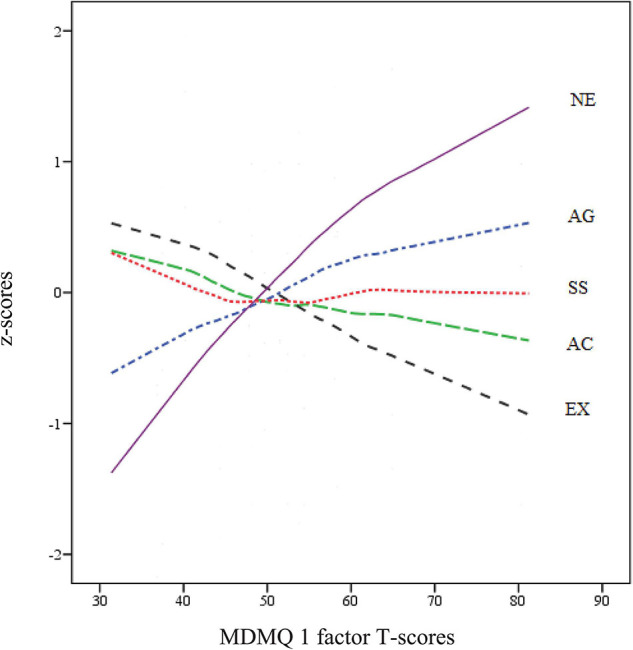
LOESS plots for ZKA-PQ/SF (z-score) and MDMQ 1 factor solution (T-score). NE = Neuroticism; AG = Aggressiveness; SS = Sensation Seeking.

Supplementary Table 3 shows the most predictive facets of each of the 4 MDMQ domains, and the prediction of each of the 5 personality domains to the Decision-Making domains. Vigilance is predicted by Work Energy (AC4) and Verbal Aggression (−AG2) (R^2^ = 0.08). Hypervigilance is positively predicted by all 4 facets of Neuroticism and, to a lesser extent, by Thrill and Adventure Seeking (−SS1) (*R*^2^ = 0.24). Buck-passing is predicted by Low Self-esteem (NE4), Work Energy (−AC4), Dependence (NE3) and Social Warmth (−EX2) (*R*^2^ = 0.18), and Procrastination is predicted by Low Self-esteem (NE4), Depression (NE2) and Work Energy (−AC4) (*R*^2^ = 0.18). The most predictive personality domains were –AG and AC (Vigilance), NE (Hypervigilance), NE (Buck-passing), and NE (Procrastination). In Figure 4, the prediction of each of the five personality domains (z-score) can be seen graphically in a non-parametric LOESS regression as the score progresses on a single decision-making factor (T-score). The most predictive domains of higher factor scores were high Neuroticism (followed by Aggressiveness) and low Extraversion (followed by Activity).

## Discussion

Our study is one of the first to investigate the relationship between decision-making and personality, using the AFFPM. The differences are partly due to the fact that the FFPM and the AFFPM have different conceptual starting points. The FFPM belongs to the lexical tradition and is a descriptive and inductively derived personality model, whereas the AFFPM draws from the temperamental personality tradition, in which basic personality constructs have strong biological foundations. They are present from birth and are observable in other species (Zuckerman, 2005). Both models include Neuroticism and Extraversion domains. The FFPM includes Agreeableness, Conscientiousness and Openness to Experience domains. The AFFPM does not consider Openness (or culture or intellect) as a personality domain but includes Activity (work compulsion and energy at work), Aggressiveness, and Sensation Seeking instead. Aggressiveness and Agreeableness dimensions have shown a strong negative association, while Activity and Conscientiousness have a positive correlation. Sensation Seeking is positively correlated with Extraversion and Openness but correlates negatively with Agreeableness and Conscientiousness (García et al., 2012).

As expected, as for the results found with the FFPM, Neuroticism and Extraversion are very positively and negatively associated with non-vigilant styles of decision-making. Note that non-vigilant styles of decision-making were placed in the Neuroticism factor in the exploratory factor analysis involving both the factors of ZKA-PQ/SF and the MDMQ. Aggressiveness is positively associated with Hypervigilance, Buck-passing, Procrastination, and negatively with Vigilance. Activity shows some low effect size correlations (around 0.10) positively with Vigilance, and negatively with, Buck-passing and Procrastination. In contrast, Sensation Seeking shows no relationship with the overall MDMQ single non-rotated factor scores and seems to have a unique pattern of correlations with four separate MDMQ domains. People high in Sensation Seeking are less sensitive to the negative consequences of their actions, make relatively riskier choices, and perform poorly on decision making tasks (Zuckerman and Kuhlman, 2000). On the other hand, people high in Neuroticism are hypersensitive to punishment and are risk-resentful (Aluja and Blanch, 2011). The combination of high Neuroticism and low Extraversion has been related to people’s vulnerability to stress and proneness to low mood and low self-esteem (Eysenck and Eysenck, 1985). Therefore, these people will tend to exhibit individual characteristics associated with non-vigilant decision-making such as hesitation, vacillation, feelings of uncertainty, and signs of emotional stress. Neuroticism and Extraversion are included in both personality models. Thus, our results are in line with the findings of other researchers using the FFPM (Di Fabio and Busoni, 2006; Di Fabio and Palazzeschi, 2012; Peter and Gurnáková, 2014; Rahaman, 2014; Heidari and Arani, 2017). Moreover, Activity shows a similar pattern of correlations with the MDMQ as does Conscientiousness in the FFPM. Aggressiveness is positively related to Hypervigilance, Buck-passing, and Procrastination, although to a lesser extent than Neuroticism.

Age was moderately negatively related to Buck-passing and Procrastination. A more detailed analysis using five age ranges between less than 30 years and more than 60 years reported that people in the youngest range score higher than the other age ranges, except the group over 60 years (Hypervigilance, Buck-passing and Procrastination). The relationship between the age ranges with the three non-vigilant decision-making styles was U-shaped, thus suggesting that young people, but also the oldest participants (to a lesser extent), tend to score higher in all three domains. This trend is found in both females and males, although females tend to score higher in all age ranges and to obtain higher overall mean scores than males in these three domains of the MDMQ. This finding is particularly interesting and contributes to a better understanding of the relationship between age and decision-making.

Another objective of this study was to examine the relationship between social position and decision-making. It was expected that people with a low social position, characterized by low educational level and occupation, would score lower on Vigilance and higher on Hypervigilance, Buck-passing and Procrastination. The results of the correlation analysis confirmed our expectations. Analysis of variance forming five categories with the SPI, clearly showed that people with better social position (lower SPI scores) scored higher on Vigilance and lower on non-vigilant styles of decision making, with relationship being clearly linear in this case. Females scored significantly higher than males in the Upper and Upper-Middle (Buck-passing), Middle and Lower-Middle (Procrastination), and in all Hypervigilance SPI categories. Note that people with a low social position were also more neurotic and less extraverted. Taking as a reference a general one-dimensional non-vigilant factor formed by the MDMQ items and considering only subjects with low and high scores on this factor, a personality profile including age and sex was obtained. According to this profile, people with high scores on this factor would tend to be young, female, low SP, aggressive, not very active, introverted, and neurotic.

## Limitations and Future Studies

This study has strengths and limitations. The main strength is the breadth of the sample, the age range, and the parity between males and females. Another positive aspect is the good structural validity and reliability of the MDMQ and ZKA-PQ/SF questionnaires. A limitation of this study is its cross-sectional design, which could compromise the validity of the results in other different contexts. A longitudinal design would have been more suitable to produce causal conclusions. Data have been collected using short self-reported questionnaires, and it would have been desirable to have interview data or parallel information on other characteristics of the sample such as psychopathological trends, perception of stress or life satisfaction. Future studies should examine the relationship between ZKA-PQ/SF with other decision-making questionnaires such as the Rational Intuitive Decision Style Scale (RI-DSS), the Proactive Decision-Making Questionnaire (PDMS) (Siebert and Kunz, 2016), and the General Decision-Making Style (GDMS) (Scott and Bruce, 1995), among others. It would also be wise to study the role of intermediate variables such as intelligence and relationship of Zuckerman’s personality model, with behavioral problems involving high impulsivity and sensation seeking traits such as gambling, alcohol abuse and dependence, or driving behavior. In addition, future studies should examine the role of SP and SES, since this variable has received little attention in relation to decision-making. Such a variable could also be assessed through more sophisticated multi-thematic indices than the Hollingshead Index of Social Position, such as the Warner’s Index of Status Characteristics or the Census Bureau’s Index of Socioeconomic Status. On the other hand, the fact that both Social Position indicators are related to IQ would require a partialling out of IQ in the correlational analysis. So, a future study should replicate those relationships between MDMQ, personality and Social Position controlling for IQ.

## Practical Implications

In regard to practical implications, the present study confirms that personality traits and, specifically, the AFFPM, as measured by the ZKA-PQ/SF, could provide useful information to help to detect and avoid difficulties in decision making, and limitations in an individual’s decision-making process. In practical and clinical contexts, therefore, personality instruments such as the ZKA-PQ/SF could be useful to support good decision-making outcomes. For instance, counselors working with people with problems in their decision-making processes should actively monitor their personality traits, with special attention to neuroticism/anxiety. They should also take into account the role of sociodemographic characteristics.

## Conclusion

Using the AFFPM, this study confirms a strong positive association of Neuroticism with Hypervigilance, Buck-passing, and Procrastination, and corresponding negative associations with Extraversion. Aggression is negatively associated with Vigilance and positively associated with Hypervigilance, Buck-passing and Procrastination. Activity is negatively associated with Buck-passing and Hypervigilance, and positively with Vigilance. People with high scores on Hypervigilance, Buck-passing and Procrastination tend to be younger and female in general, but an examination of five age ranges shows that those over 60 tend to score comparably to those aged 30 and younger. Females score lower in Vigilance and higher in non-vigilant styles of decision-making. Low SP is associated with low scores in Vigilance and high scores in non-vigilant styles of decision-making.

## Data Availability Statement

The original contributions presented in the study are included in the article/Supplementary Material, further inquiries can be directed to the corresponding author.

## Ethics Statement

The studies involving human participants were reviewed and approved by Comitè d’Ètica Hospital Universitari Arnau de Vilanova de Lleida (Spain). The patients/participants provided their written informed consent to participate in this study.

## Author Contributions

All authors listed have made a substantial, direct and intellectual contribution to the work, and approved it for publication.

## Conflict of Interest

The authors declare that the research was conducted in the absence of any commercial or financial relationships that could be construed as a potential conflict of interest.

## Publisher’s Note

All claims expressed in this article are solely those of the authors and do not necessarily represent those of their affiliated organizations, or those of the publisher, the editors and the reviewers. Any product that may be evaluated in this article, or claim that may be made by its manufacturer, is not guaranteed or endorsed by the publisher.
