# Supplementary material for: Decision-Making and the Alternative Five Factor Personality Model: Exploring the Role of Personality Traits, Age, Sex and Social Position
Source: Front Psychol. 2021 Oct 27;12:717705. doi: 10.3389/fpsyg.2021.717705 (PMC8578866; doi:10.3389/fpsyg.2021.717705)
Supplement: Supplementary file 1 [file Table_1.DOCX]

Table S-1. *Frequency and percentages of sex (n = 1,562) and sociodemographic variables (n = 1,006).*

| **Sex** | Frequency | % total sample | % Community sample |
| --- | --- | --- | --- |
| Male | 714 | 45.7 | -- |
| Female | 848 | 54.3 | -- |
| *Total sample* | *1,562* | *100.0* | *--* |
| **Studies** |  |  |  |
| 1-University degree | 236 | 15.1 | 32.1 |
| 2-University school, no faculty | 292 | 18.7 | 8.9 |
| 3-Some university course (faculty) | 174 | 11.1 | 3.1 |
| 4-High School or Certificate of Higher Education (HNC) | 109 | 7.0 | 25.1 |
| 5-Some high school or similar course. | 94 | 6.0 | 10.0 |
| 6-Secondary School. | 28 | 1.8 | 13.6 |
| 7-Some Primary or secondary course | 73 | 4.7 | 7.1 |
| *Total* | *1,006* | *64.4* | *100* |
| **Profession** |  |  |  |
| 1-Large company / Professional / senior executive | 323 | 20.7 | 23.5 |
| 2-Average company / Average professional | 90 | 5.8 | 29.0 |
| 3-Small businesses / Skilled worker | 31 | 2.0 | 17.3 |
| 4-Office worker / seller | 253 | 16.2 | 10.8 |
| 5-Specialized manual workers | 101 | 6.5 | 9.3 |
| 6-Semi-specialized manual workers | 137 | 8.8 | 2.8 |
| 7-Unskilled workers | 71 | 4.5 | 7.3 |
| *Total* | *1,006* | *64.4* | *100* |
| **Social Position Index ranges** |  |  |  |
| 1.-Upper (11-17) | 189 | 12.1 | 18.8 |
| 2.-Upper-middle (18-31) | 328 | 21.0 | 32.6 |
| 3.-Middle (32-47) | 252 | 16.1 | 25.0 |
| 4.-Lower-middle (48-63) | 145 | 9.3 | 14.4 |
| 5.-Lower (64-77) | 92 | 5.9 | 9.1 |
| *Total* | *1,006* | *64.4* | *100* |

*Note:* The highest SPI values ​​correspond to the lowest social position.

Table S-2. *MDMQ Procrustes factor matrix and factor congruence coefficients*

*between community and university students samples.*

| Item | F-I | F-II | F-III | F-IV | *I.C*. |
| --- | --- | --- | --- | --- | --- |
| 1 | **.58** | .02 | -.04 | .07 | *1* |
| 2 | **.48** | -.09 | .06 | -.02 | *.97* |
| 3 | **.65** | .18 | .08 | .03 | *.98* |
| 4 | **.55** | -.06 | -.04 | -.01 | *1* |
| 5 | **.48** | .07 | -.09 | -.10 | *.99* |
| 6 | **.51** | -.21 | .04 | -.08 | *.99* |
| 7 | -.16 | **-.63** | .03 | -.03 | *.97* |
| 8 | .00 | **-.56** | .06 | .06 | *.99* |
| 9 | .09 | **-.67** | -.02 | -.02 | *1* |
| 10 | .02 | **-.39** | .25 | .03 | *.96* |
| 11 | .05 | **-.39** | .00 | .12 | *.96* |
| 12 | -.04 | .00 | **.58** | .27 | *1* |
| 13 | -.03 | -.09 | **.54** | .24 | *.98* |
| 14 | -.07 | .04 | **.74** | .08 | *1* |
| 15 | -.07 | -.16 | **.60** | .07 | *1* |
| 16 | -.10 | .05 | **.69** | -.07 | *.96* |
| 17 | .14 | -.11 | **.29** | -.08 | *.92* |
| 18 | .05 | **-.43** | -.09 | **.22** | *.94* |
| 19 | -.04 | -.23 | -.09 | **.52** | *.99* |
| 20 | .02 | .01 | .02 | **.66** | *.99* |
| 21 | -.09 | -.06 | .01 | **.64** | *.99* |
| 22 | .03 | .19 | .14 | **.72** | *1* |
| *C.C.* | *.98* | *.97* | *.99* | ***.98*** | *.98* |

*Note: MDMQ: Melbourne Decision Making Questionnaire. F-I: Vigilance; F-II; Hypervigilance; F-III: Buck-passing; F-IV; Procrastination; I.C.: Item congruence. Congruency Coefficients.* Absolute value loadings equal or higher .30 in boldface.

Table S-3. *Multiple Linear Regression Models.*

| ZKA-PQ/SF facets (independent variable) and MDMQ domains (dependent variable). | | | | | | | |
| --- | --- | --- | --- | --- | --- | --- | --- |
| Vigilance | β | Hypervigilance | β | Buck-passing | β | Procrastination | β |
| AC4 Work Energy | .221 | NE2 Depression | .151 | NE4 Low Self-esteem | .255 | NE4 Low Self-esteem | .210 |
| AG2 Verbal Aggression | -.121 | NE4 Low Self-esteem | .200 | AC4 Work Energy | -.104 | NE2 Depression | .181 |
|  |  | NE3 Dependence | .153 | NE3 Dependence | .134 | AC4 Work Energy | -.138 |
|  |  | NE1 Anxiety | .171 | EX2 Social Warmth | -.100 |  |  |
|  |  | SS1Thrill and Adventure Seeking | -.120 |  |  |  |  |
| *R = .28; R^2^ = 07* |  | *R = .49; R^2^ = .24* |  | *R = .43; R^2^ = .18* |  | *R = .42; R^2^ = .18* |  |
|  |  | ZKA-PQ/SF domains (independent variable) and MDMQ domains (dependent variable). | | | | |  |
| Vigilance | β | Hypervigilance | β | Buck-passing | β | Procrastination | β |
| Aggressiveness | -.154 | Aggressiveness | .008 | Aggressiveness | -.054 | Aggressiveness | .024 |
| Activity factor | .122 | Activity factor | .042 | Activity factor | -.094 | Activity factor | -.087 |
| Extraversion | .023 | Extraversion | -.059 | Extraversion | -.098 | Extraversion | -.090 |
| Neuroticism | -.020 | Neuroticism | .560 | Neuroticism | .369 | Neuroticism | .364 |
| Sensation Seeking | -.077 | Sensation Seeking | -.113 | Sensation Seeking | .004 | Sensation Seeking | .053 |
| *R = .23; R^2^ = .05* |  | *R = .59; R^2^ = .35* |  | *R = .41; R^2^ = .17* |  | *R = .43; R^2^ = .18* |  |

Note: ZKA-PQ/SF: Zuckerman-Kuhlman-Aluja Personality Questionnaire shortened form; MDMQ: Melbourne Decision Making Questionnaire. β: Beta.
